# 

**Published:** 1890-04-12

**Authors:** 


### NEW OFFICES IN THE STRAND.

The Hospital *is now published at* **140, Strand, W.C.** *It has been found desirable to establish* The Hospital *in this central and convenient locality, owing to the rapid extension of the business of this paper of late.*

### NOTICE TO CORRESPONDENTS.

All MS., Letters, Books for Review, and other matters intended for the Editor should be addressed The Editor, The Lodge, Porchester Square, London, W.

The Editor cannot undertake to return rejected M.S., even when accompanied by stamped directed envelope.

**Notice to Advertisers and Subscribers.**

All Advertisements, Orders for Copies of The Hospital, and Business Communications should be addressed to The Publisher, *not to the Editor*, The Hospital, 140, Strand, W.C.

**Bound Volumes of "The Hospital."**

Vol. VI., containing full page portraits of T.R.H. the Prince and Princess of Wales, and Miss Florence Nightingale, is now ready, 4s. post free. Cases for binding may be had of the Publisher, at 1s. 6d. each.

**To Residents, Secretaries, and Matrons.**

The Editor will be glad to have the Regulations and each Report as issued by Asylums, Hospitals, Convalescent and Nursing Institutions, as well as those of other Charities, and an early copy in duplicate of all Appeals and Festival Notices, etc., addressed to *The Editor, The Lodge, Porchester Square, London, W.* Any superintendent, secretary, matron, or other chief official who regularly sends such information may be registered, on application to the Publisher, to receive free of cost a cover for binding The Hospital in half-yearly volumes.

### Scraps and Gleanings.

The Queen is undergoing a course of massage at Aix.

Mr. E. Ward has been appointed house surgeon to Reading Hospital.

At the Hull and Sulcotes Dispensary, an average of 198 patients attend each day.

Exeter Dental Hospital treated 4,398 cases last year, being an increase of 300.

Mr. Hubert Higgins has been appointed house surgeon to Addenbrooke's Hospital.

A Samaritan Fund is to be established in connection with the Worcester Infirmary.

It is proposed to again enlarge the Princess Alice Memorial Hospital to hold another ten beds.

Scarborough Hospital and Dispensary treated 368 in-patients last year, at a total cost of £1,091.

The Duke of Westminster presided at the annual meeting of the Alexandra Children's Hospital at Rhyl.

A committee has been formed to manage the Chester Skin Dispensary, founded last November by Dr. Roberts.

Ilkley Hospital and Convalescent Home treated 1,084 cases last year; the working expenses were £1,659.

The City of Dublin Hospital treated over 1,000 in, and over 14,000 out-patients last year. It reports a deficit.

Black Diphtheria has broken out at Wilkesbarre (Pa.), and the mortality amongst children has been enormous.

Lord Bristol presided at the General Board of the Suffolk Hospital, when the new rules were discussed and approved.

A despatch from Hudson's Bay says the Indians of that territory are dying of starvation; women kill and eat their children.

Dr. McCullagh has been lecturing at St. Mark's Hospital, Dublin, to the Ladies' Sanitary Association, on the subject of "Light."

The deaths from diphtheria in the metropolitan area are just now much above the average. The deaths primarily attributed to influenza have declined to 13.

At Montreal the Canadian Women's Board of Missions have considered and decided upon the necessity of sending two ladies as missionaries to the Ahmad Nagar district.

Madame Carnot has visited the Day Nursery in the Rue Bacon, kept by the Sisters of Charity. There are 70 cots, and each cot is endowed in memory of some lost child.

Bolton people are great supporters of the St. John Ambulance Association; 34 ladies have just taken nursing certificates, and two classes for men have just been started.

A "temperance exhibition" will shortly be opened at the Agricultural Hall. Its scheme includes model homes, this portion of the display being directed by the Salvation Army.

Berwick Dispensary and Infirmary issues a satisfactory report so far as funds are concerned. The treasurer is not likely to be "inundated" with magazines after his late statement.

A traveller, Caleb Henry Jones, while undergoing an operation for some skin disease at Swansea, died suddenly during the administration of a mixture of alcoholic chloroform and ether.

Rosalie, the ape which the Princess Imperial of Brazil sent to Dr. Charcot as a token of gratitude for the medical services which he rendered to her father, has just died of inflammation of the lungs.

The Duchess of Fife will open the grand fancy bazaar on Wednesday, June 4, at 12 noon, in aid of the funds of University College Hospital. The bazaar will be held in the grounds of University College, Gower Street.

Sir Guyer Hunter has been appointed Chairman of the Committee of Inquiry into the Water-supply of the City and its cost. The inquiry will be watched with interest by more than those who use the City water-supply.

Mrs. Margaret Rea, of Lylehill, county Down, died at her residence, on Saturday, from an attack of influenza. She was a hundred years old, had been a widow over fifty years, and was in full possession of her mental faculties to the last.

The Royal Medical Benevolent College has received a legacy of £400 from the late Mrs. Price, of Leamington, and also £250 assigned to it by the trustees of the late Mr. Daniel Procter, of Manchester, who left a large residue at their disposal for charitable purposes.

A Chinese laundryman has been admitted to the Philadelphia Hospital suffering from leprosy. It is feared that several other cases exist, as many people have been exposed to the chances of catching it from the fact that Hop Yan Lee has been doing his daily quantity of washing.

Wells Cottage Hospital held its annual meeting on March 26th, the president and founder of the institution, the bishop of the diocese, in the chair. The report was in every way satisfactory, as 49 cases were treated, and all more or less relieved; and there is a balance of £24 to carry forward to next year.

Australian mail news states that Miss E. Constance Stone, who was assistant physician at the New Hospital for Women in London, has just been registered by the Melbourne Medical Board as a medical practitioner, being the first Australian lady doctor who has been granted registration in the Australian colonies.

